# Anti-PD-1 and Anti-CTLA-4 Therapies in Cancer: Mechanisms of Action, Efficacy, and Limitations

**DOI:** 10.3389/fonc.2018.00086

**Published:** 2018-03-28

**Authors:** Judith A. Seidel, Atsushi Otsuka, Kenji Kabashima

**Affiliations:** ^1^Department of Dermatology, Kyoto University Graduate School of Medicine, Kyoto, Japan; ^2^Singapore Immunology Network (SIgN), Institute of Medical Biology, Agency for Science, Technology and Research (A*STAR), Biopolis, Singapore, Singapore

**Keywords:** immunotherapy, cancer, melanoma, side effects, biomarkers, immune checkpoint inhibitors, mode of action

## Abstract

Melanoma, a skin cancer associated with high mortality rates, is highly radio- and chemotherapy resistant but can also be very immunogenic. These circumstances have led to a recent surge in research into therapies aiming to boost anti-tumor immune responses in cancer patients. Among these immunotherapies, neutralizing antibodies targeting the immune checkpoints T-lymphocyte-associated protein 4 (CTLA-4) and programmed cell death protein 1 (PD-1) are being hailed as particularly successful. These antibodies have resulted in dramatic improvements in disease outcome and are now clinically approved in many countries. However, the majority of advanced stage melanoma patients do not respond or will relapse, and the hunt for the “magic bullet” to treat the disease continues. This review examines the mechanisms of action and the limitations of anti-PD-1/PD-L1 and anti-CTLA-4 antibodies which are the two types of checkpoint inhibitors currently available to patients and further explores the future avenues of their use in melanoma and other cancers.

## Introduction

In recent years, there has been a steep rise in the development and implementation of anti-cancer immunotherapies. The approval of anti-cytotoxic T-lymphocyte-associated protein 4 (CTLA-4) and anti-programmed cell death protein 1 (PD-1) antibodies for human use has already resulted in significant improvements in disease outcomes for various cancers, especially melanoma. Unlike radio- and chemotherapy, which aim to directly interfere with tumor cell growth and survival, immunotherapies target the tumor indirectly by boosting the anti-tumor immune responses that spontaneously arise in many patients.

## Cancers Evade and Inhibit Immune Responses

In order to understand the modes of action of immune checkpoint inhibitors, it is important to understand the dynamic interplay between cancers and the immune system during the course of the disease.

Cancer cells are genetically unstable, which contributes to their uncontrolled proliferation and the expression of antigens that can be recognized by the immune system. These antigens include normal proteins overexpressed by cancer cells and novel proteins that are generated by mutation and gene rearrangement (1). Cytotoxic CD8^+^ T cells are immune cells that are particularly effective at mediating anti-tumor immune responses. These cells may learn to recognize the tumor-specific antigens presented on major histocompatibility complex (MHC) class I molecules and thereby perform targeted tumor cell killing. CD8^+^ T cells become licensed effector cells after appropriate stimulation by antigen-presenting cells that have collected antigens at the tumor site. Apart from the antigen peptides embedded on the MHC molecules, antigen-presenting cells must provide costimulatory signals through surface receptors (such as CD28) and cytokines [such as interleukin (IL)-12] for effective T cell stimulation (2).

Tumor cells adopt a variety of mechanisms to avoid immune recognition and immunomediated destruction. Established tumors are often thought to arise through the selection of clones that are able to evade the immune system, a process known as immunoediting (3). Tumor cells may evade immune recognition directly by downregulating features that make them vulnerable such as tumor antigens or MHC class I (4–6). Alternatively, tumors may evade immune responses by taking advantage of negative feedback mechanisms that the body has evolved to prevent immunopathology. These include inhibitory cytokines such as IL-10 and tumor growth factor (TGF)-β, inhibitory cell types such as regulatory T cells (Tregs), regulatory B cells (Bregs), and myeloid-derived suppressor cells (MDSCs), metabolic modulators such as indoleamine 2,3-dioxygenase (IDO), and inhibitory receptors such as PD-1 and CTLA-4 (7, 8).

## Immune Exhaustion Contributes to Immune Dysfunction in Cancer

Inhibitory receptors, also known as immune checkpoints, and their ligands can be found on a wide range of cell types. They are essential for central and peripheral tolerance in that they counteract simultaneous activating signaling through co-stimulatory molecules. Inhibitory receptors may act during both immune activation and ongoing immune responses. During chronic inflammation in particular, T cells are known to become exhausted and to upregulate a wide range of non-redundant inhibitory receptors that limit their effectiveness, such as PD-1, CTLA-4, T-cell immunoglobulin and mucin-domain containing-3 (TIM-3), lymphocyte-activation gene 3 (LAG-3), or T-Cell immunoreceptor with Ig And ITIM domains (TIGIT) [See Table 1 (9–11)]. Originally described in the context of chronic viral infections, where the host fails to clear the pathogen, it is now apparent that exhausted T cells can also occur in cancer (12, 13). It is believed that, under these conditions, persistent high antigenic load leads to the T cells upregulating the inhibitory receptors, whose signaling subsequently leads to a progressive loss of proliferative potential and effector functions and in some cases to their deletion (14).

**Table 1:** Overview of T cell surface receptors associated with immune inhibition and dysfunction.

Receptor	Expressing cells	Ligands	Ligand-expressing cells
Programmed cell death protein 1 (PD-1) (11)	CD4 (activated/exhausted, follicular), CD8 (activated/exhausted), B cells, dendritic cells (DCs), monocytes, mast cells, Langerhans cells	PD-L1, PD-L2	Antigen-presenting cells, CD4^+^ T cells, non-lymphoid tissues, some tumors

T-lymphocyte-associated protein 4 (CTLA-4) (15)	CD4 (activated/exhausted, Tregs), CD8 (activated/exhausted), some tumors	CD80, CD86	Antigen-presenting cells

lymphocyte-activation protein 3 (LAG-3) (15)	CD4 (including Treg and exhausted), CD8 (including exhausted), natural killer cells (NK)	MHC class II, LSECtin	Antigen-presenting cells, liver, some tumors

T-cell immunoglobulin and mucin-domain containing-3 (TIM-3) (16)	CD4 (Th1, Th17, Treg), CD8 (including exhausted and Tc1), DC, NK, monocyte, macrophages	Galectin-9, phosphatidyl serine, high mobility group protein B1, Ceacam-1	Endothelial cells, apoptotic cells, some tumors

T-cell immunoreceptor with Ig And ITIM domains (TIGIT) (16)	CD4 (including Treg, follicular helper T cells), CD8, NK	CD155 (PVR), CD122 (PVRL2, nectin-2)	APCs, T cells, some tumors

Exhaustion is therefore both a physiological mechanism designed to limit immunopathology during persistent infection and a major obstacle for anti-tumor immune responses (17). It should be noted that expression of inhibitory markers is not always a sign of immune exhaustion, because the receptors may be expressed individually during conventional immune responses (18).

## The Immune Checkpoint Receptor CTLA-4

The anti-CTLA-4 blocking antibody ipilimumab was the first immune checkpoint inhibitor to be tested and approved for the treatment of cancer patients (19, 20). CTLA-4 (CD152) is a B7/CD28 family member that inhibits T cell functions. It is constitutively expressed by Tregs but can also be upregulated by other T cell subsets, especially CD4^+^ T cells, upon activation (21). Exhausted T cells are also often characterized by the expression of CTLA-4 among other inhibitory receptors. CTLA-4 is mostly located in intracellular vesicles and is only transiently expressed upon activation in the immunological synapse before being rapidly endocytosed (22).

CTLA-4 mediates immunosuppression by indirectly diminishing signaling through the co-stimulatory receptor CD28. Although both receptors bind CD80 and CD86, CTLA-4 does so with much higher affinity, effectively outcompeting CD28 (23). CTLA-4 may also remove CD80 and CD86 (including their cytoplasmic domains) from the cell surfaces of antigen-presenting cells *via* trans-endocytosis (24), therefore reducing the availability of these stimulatory receptors to other CD28-expressing T cells. Indeed, this process is an important mechanism by which Tregs mediate immune suppression on bystander cells (25).

By limiting CD28-mediated signaling during antigen presentation, CTLA-4 increases the activation threshold of T cells, reducing immune responses to weak antigens such as self- and tumor antigens. The central role that CTLA-4 plays in immunological tolerance is exemplified by experiments in mice that lack the CTLA-4 gene globally or specifically in the Forkhead box P3 (FoxP3)^+^ Treg compartment. These animals develop lymphoproliferative disorders and die at a young age (25, 26). Similarly, polymorphisms within the CTLA-4 gene are associated with autoimmune diseases in humans (27). CTLA-4 signaling has been shown to dampen immune responses against infections and tumor cells (28, 29).

## The Immune Checkpoint Receptor PD-1

The surface receptor PD-1 (CD279) was first discovered on a murine T cell hybridoma and was thought to be involved in cell death (30). It has since become clear, however, that PD-1, which is homologous to CD28, is primarily involved in inhibitory immune signaling, and is an essential regulator of adaptive immune responses (31). In both humans and mice some T cell populations constitutively express PD-1; one example is follicular helper T cells (32). Although most circulating T cells do not express the receptor, they can be induced to do so upon stimulation, through the T cell receptor (TCR) complex or exposure to cytokines such as IL-2, IL-7, IL-15, IL-21, and transforming growth factor (TGF)-β (33, 34). Other cell types, such as B cells, myeloid dendritic cells, mast cells, and Langerhans cells, can also express PD-1 which may regulate their own and bystander cell functions under pathophysiological conditions (35–38). PD-1 has two ligands: PD-L1 (B7-H1; CD274) and PD-L2 (B7-DC; CD273). Both can be found on the surface of antigen-presenting cells (such as dendritic cells, macrophages, and monocytes), but are otherwise differentially expressed on various non-lymphoid tissues (39, 40). Interferon (IFN)-γ is the main trigger known to cause PD-L1 and PD-L2 upregulation (41).

**Figure 1:**
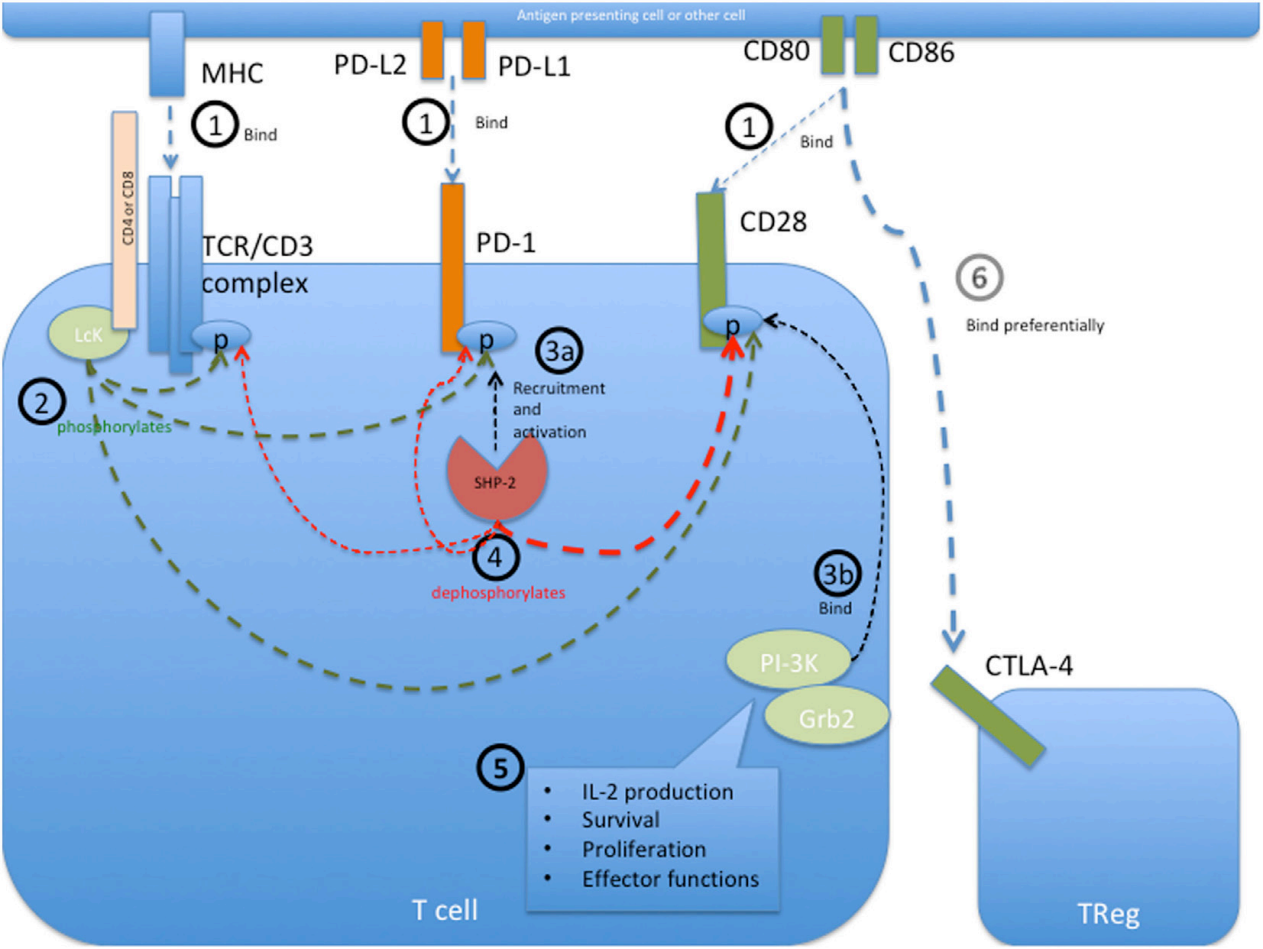
Programmed cell death protein 1 (PD-1) mediated intracellular signaling events during T cell activation. **(1)** Upon T cell activation, the extracellular receptors PD-1, CD28, and the T cell receptor (TCR) complex (including CD4 or CD8) bind their ligands PD-L1 or PD-L2, CD80 or CD86, and major histocompatibility complex (MHC) class I or II, respectively. This brings all the receptors into close proximity with each other at the immunological synapse and allows them to interact with each other. **(2)** The Src kinase Lck (P56Lck), which is bound to the intracellular tail of CD4 and CD8, can now phosphorylate the tyrosine residues on the intracellular tails of PD-1 and CD28 as well as the CD3ζ chain of the TCR/CD3 complex. **(3a)** Phosphorylation of the immunoreceptor tyrosine-based switch motif (ITSM) motif on the intracellular tail of PD-1 allows recruitment of the Src homology region 2 domain-containing phosphatase 2 (SHP-2), resulting in the activation of SHP-2 phosphatase activity. SHP-1 may also bind PD-1 but to a lesser extent than SHP-2. **(3b)** Simultaneously, the phosphorylated tail of CD28 is now able to recruit PI-3K and Grb2 among other signaling molecules. **(4)** Through close proximity at the immunological synapse, PD-1-associated SHP-2 can dephosphorylate the cytoplasmic tail of CD28, and to a lesser extent that of the CD3ζ chain, therefore preventing the recruitment of further downstream signaling molecules associated with these molecules. SHP-2 may also dephosphorylate PD-1, causing auto-regulation of this inhibitory pathway. **(5)** CD28 provides critical signals alongside TCR stimulation, and the abrogated binding of PI3K and Grb2 to this receptor therefore leads to decreased signaling in pathways important for IL-2 production, survival, proliferation, and certain effector functions. In the absence of its ligands, PD-1 is not recruited to the immune synapse and can therefore not interfere with activation signaling. **(6)** The inhibitory receptor CTLA-4 primarily restricts CD28 signaling indirectly by reducing the availability of CD80 and CD86, to which it binds with a much higher affinity than the co-stimulatory receptor CD28. Sources (43–45).

PD-1 bears an immunoreceptor tyrosine-based inhibition motif (ITIM) and an immunoreceptor tyrosine-based switch motif (ITSM) motif on its intracellular tail. The intracellular signaling events initiated upon PD-1 engagement are best described in T cells and are illustrated in Figure 1. In these cells, engagement of PD-1 causes tyrosine residues to become phosphorylated, starting an intracellular signaling cascade that mediates the dephosphorylation of TCR proximal signaling components (9, 42–44). Among these, CD28 has recently been found to be the primary target (45). In the presence of TCR stimulation, CD28 provides critical signals that are important for T cell activation. By interfering with early TCR/CD28 signaling and associated IL-2-dependent positive feedback, PD-1 signaling therefore results in reduced cytokine production [such as IL-2, IFN-γ, and tumor necrosis factor (TNF)-α], cell cycle progression, and pro-survival Bcl-xL gene expression, as well as reduced expression of the transcription factors involved in effector functions such as T-bet and Eomes (42, 43, 46, 47). PD-1 activity is therefore only relevant during simultaneous T cell activation, as its signal transduction can only come into effect during TCR-dependent signaling (39, 41, 48). Details about PD-1 signaling in other cell types that bear this receptor, such as B cells, remain to be elucidated.

Overall, PD-1 is crucial for the maintenance of peripheral tolerance and for containing immune responses to avoid immunopathology. Mice deficient in the receptor initially appear healthy, but develop autoimmune diseases such as lupus-like proliferative glomerulonephritis and arthritis with age and exacerbated inflammation during infections (18, 31, 49, 50). Humans with genetic polymorphisms in the PD-1 locus also have an increased likelihood of developing various autoimmune diseases (51, 52).

## CTLA-4, PD-1, and Their Ligands in Cancer

CTLA-4 may be expressed in tumor lesions on infiltrating Tregs or exhausted conventional T cells as well as tumor cells themselves (53, 54). Despite the immunosuppressive role of CTLA-4, its association with disease prognosis is not clear; however, it should be noted that only a few studies have described the prognostic value of CTLA-4 levels in the tumor site. So far, the expression of CTLA-4 on tumors has been associated with decreased survival in nasopharyngeal carcinoma (54) and increased survival in non-small cell lung cancer (53).

PD-1 can be upregulated transiently during stimulation and constitutively during chronic immune activation (17). The inhibitory receptor has been detected on both circulating tumor-specific T cells and tumor-infiltrating lymphocytes, where it was associated with decreased T cell function in humans and mice (13, 29, 55–57). Other cell types may also upregulate PD-1 in tumor lesions. PD-1-positive dendritic cells, for example, have been identified in hepatocellular carcinoma where they exhibited a reduced ability to stimulate T cells (37). Another study identified a population of tumor-infiltrating PD-1-expressing regulatory B cells that produced IL-10; higher proportions of these cells were correlated with worse disease outcome in hepatocellular carcinoma patients (58). Tumor-associated macrophages were also recently shown to express PD-1 in both mice and humans with colorectal cancer and to impair macrophage phagocytosis (59).

Both cancer cells and tumor-infiltrating immune cells (such as macrophages) may express PD-L1 and upregulate it in response to IFN-γ (60). PD-L1 expression may therefore be indicative of active anti-tumor immune responses and may also actively contribute to local immunosuppression. The relationship between PD-1 or PD-L1 expression at the tumor site and disease outcome is thus not consistent among all tumor types and patients. High PD-1 and/or PD-L1 may correlate with poor prognosis in some cancers (including melanoma, renal cell carcinoma, esophageal, gastric, and ovarian cancers) and with improved prognosis in others (such as angiosarcoma and gastric cancer) (55, 60–65).

## Efficacy and Mode of Action of Checkpoint Inhibitors

Both CTLA-4 and PD-1 checkpoint inhibitors have resulted in increased patient survival in a number of studies, including studies on melanoma, renal cell carcinoma, squamous cell carcinoma, and non-small cell lung cancer, when compared to conventional chemotherapies (summarized in Table 2). In melanoma, anti-PD-1 treatment was more effective in patients with smaller tumors (66). A direct comparison between the two checkpoint inhibitors in a Phase III clinical trial found better response (44%) and survival rates (6.9 months progression-free survival) among patients treated with the anti-PD-1 antibody nivolumab than among those treated with the anti-CTLA-4 antibody ipilimumab (19% and 2.8 months). Combined administration of both nivolumab and ipilimumab resulted in even higher response rates (58%) and survival (11.5 months) (67).

**Table 2:** Treatment outcome of clinical trials for immune checkpoint inhibitors in various cancer types.

Target		Drug	Condition	Treatment regimen	Treatment in control group	Objective response rate	Complete response rates	Overall survival (months)	Progression-free survival (months)	Grade 3–5 adverse events	Participants treated (and controls)	Reference
Programmed cell death protein 1 (PD-1) signaling	PD-1	Nivolumab (IgG4a)	Melanoma (stage III/IV)	3 mg/kg/2 weeks	(*vs* combination therapy)	43.7%	8.9%	n/a	6.9	16.3%	316	(67)

			Renal cell carcinoma (metastatic)	3 mg/kg/2 weeks	10 mg/day Everolimus	25% (4% control)	1% (<1% control)	25.0 (19.6 control)	4.6 (4.4 control)	19% (27% control)	406 (397 control)	(68)

			Hodgkin’s lymphoma (relapsed/refractory)	3 mg/kg/2 weeks	n/a	87%	17%	n/a	86% at 24 weeks	22%	23	(69)

			Squamous-cell carcinoma of the head and neck (recurrent)	3 mg/kg/2 weeks	Single-agent systemic therapy (methotrexate, docetaxel, or cetuximab)	13.3% (5.8% control)	2.5% (0.8% control)	36.0%/1 year (16.6% control)	19.7% at 6 months (9.9% control)	13.1% (35.1%)	240 (121 control)	(70)

			Non-small cell lung cancer	3 mg/kg/2 weeks	Docetaxel	19% (12% control)	1% (<1% control)	12.2 (9.4 control)	2.3 (4.2 control)	10% (54% control)	292 (290 control)	(71)

				3 mg/kg/2 weeks	Docetaxel	20% (9% control)	1% (0% control)	9.2 (6 control)	3.5 (2.8 control)	7% (55% control)	135 (137 control)	(72)

			Ovarian cancer (platinum-resistant)	1 or 3 mg/kg/2 weeks	n/a	15%	10%	20	3.5	40%	20	(62)

		Pembrolizumab (IgG4a)	Melanoma (stage III/IV)	10 mg/2 weeks or 3 weeks	(*vs* ipilimumab)	33.7–32.9%	5.0–6.1%	n/a	5.5–4.1	13.3–10.1%	279–277	(73)

			Merkel cell carcinoma	2 mg/kg/3 weeks	n/a	56%	16%	n/a	65% at 6 months	15%	26	(74)

			Non-small cell lung cancer	2 mg/kg/3 weeks 10 mg/kg/3 weeks 10 mg/kg/2 weeks	n/a	19.4%	n/a	12	3.7	9.5%	495	(75)

				200 mg/2 weeks (PD-L1 + patients only)	Platinum-based chemotherapy	44.8 (27.8% control)	n/a	80.2% at 6 months (72.4% control)	10.3 (6 control)	26.6% (53.3% control)	154 (154 control)	(76)

				2 or 10 mg/kg/3 weeks (PD-L1 + patients only)	Docetaxel	18/18% (9% control)	0/0% (0% control)	10.4/12.7 (8.5 control)	3.9/4.0 (4.0 control)	13/16% (35% control)	345/346 (343 control)	(77)

			Progressive metastatic colorectal cancer	10 mg/kg/every 2 weeks	n/a	40/0%	0/0%	>5 months/5	>5/2.2	41% overall	10/18	(78)

		Pidilizumab (IgG1)	B cell lymphoma (after autologous stem cell transfer)	1.5 mg/42 days	n/a	51%	34%	85% at 16 months	72% at 16 months	n/a	66	(79)

			Follicular lymphoma (relapsed)	3 mg/kg/4 weeks (+ rituximab)	n/a	66%	52%	n/a	n/a	0%	29	(80)

	PD-L1	Atezolizumab (IgG1)	Non-small cell lung cancer (stage III–IV)	1,200 mg/3 weeks	Docetaxel	18% (16% control)	2% (<1% control)	15.7 (10.3 control)	2.8 (4 control)	15% (43% control)	425 (425 control)	(81)

			Urothelial carcinoma (locally advanced and metastatic)	1,200 mg/3 weeks	n/a	23%	9%	15.9%	2.7	16%	119	(82)

T-lymphocyte-associated protein 4 (CTLA-4) signaling	CTLA-4	Ipilimumab (IgG1)	Melanoma (stage III/IV)	10 mg/kg plus decarbazine	Decarbazine alone	15.2% (10.3% control)	1.6% (0.8% control)	11.2 (9.1 control)	n/a	56.3% (27.5%)	250 (252 control)	(83)

				3 mg/kg/3 weeks	(*vs* Pembrolizumab)	11.9%	1.4%	n/a	2.8	19.9%	278315	(73)

				3 mg/kg/3 weeks	(*vs* combination with nivolumab)	19%	2.2%	n/a	2.9	27.3%	311	(67)

		Tremelimumab (IgG2)	Melanoma (stage III/IV)	15 mg/kg/90 days	chemotherapy (temozolomide or dacarbazine)	10.7% (9.8% control)	3% (2% control)	12.6% (10.7 control)	20.3% at 6 months (18.1% control)	52% (37% control)	328 (327 control)	(84)

Combination therapy		Nivolumab + Ipilimumab	Melanoma (stage III/IV)	3 mg/kg/2 weeks Nivolumab 3 mg/kg/3 weeks Ipilimumab	(*vs* single)	57.6%	11.5%	n/a	11.5	55%	314	(67)

			Non-small cell lung cancer	Nivo + Ipi: 1 + 3 or 3 + 1 mg/ml	Nivolumab alone	23/19% (10% control)	2/0% (0%)	7.7/6 (4.4)	2.6/1.4 (1.4 control)	30/19% (13% control)	61/54 (98 control)	(85)

*n/a: not available*.

*Where the median values for overall or progression-free survival were not reached within the time frame of a study and the percentage of patients surviving for a given time frame are shown instead*.

*The anti-PD-L1 antibodies avelimumab and durvalumab are currently undergoing early-stage clinical trials and therefore no data has yet been published on their efficacy*.

Both CTLA-4 and PD-1 act independently as brakes on CD3/CD28-dependent signaling, suggesting that underlying immune responses are required for checkpoint inhibitor treatment to take effect (66). Indeed, as mentioned in the previous section, both PD-1 and CTLA-4 blockades are more effective in tumors that are infiltrated by T cells or that have high mutation rates and are therefore more immunogenic prior to treatment (86–88).

**Figure 2:**
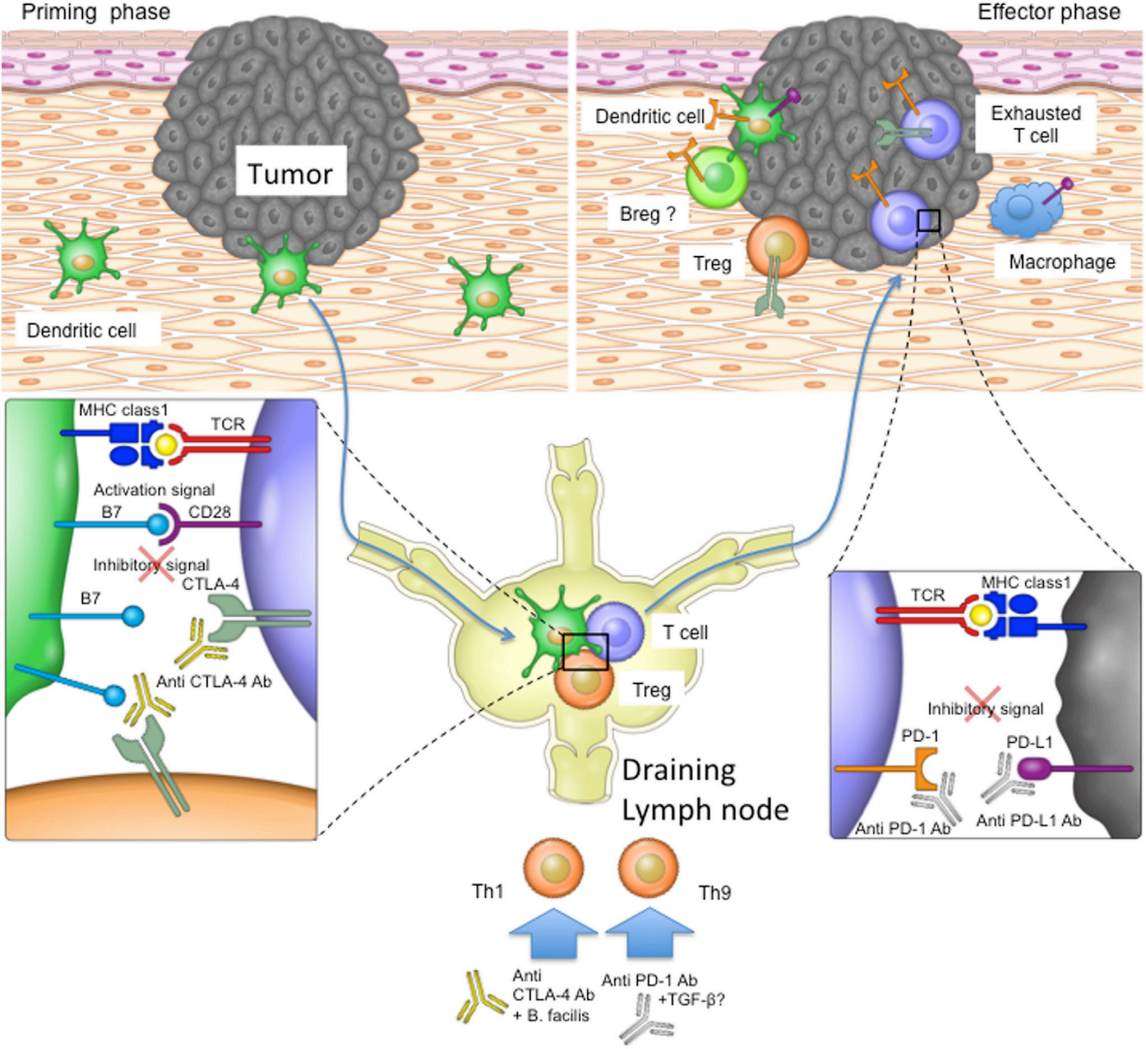
The role of programmed cell death protein 1 (PD-1) and T-lymphocyte-associated protein 4 (CTLA-4) in the priming and effector phases of anti-tumor immune responses. For T cell priming, dendritic cells (DCs) sample antigen at the tumor site and transport it to the draining lymph nodes, where they present the antigens on their major histocompatibility complex (MHC) molecules to T cells. T cells become activated if their T cell receptors recognize and bind the antigen on MHC complexes and their CD28 costimulatory receptors bind CD80 and CD86 on DCs. CTLA-4 upregulation on T cells or bystander Tregs can interfere with the CD28 signal, as the former receptor binds CD80 and CD86 with higher affinity. Once activated, T cells migrate to the tumor site in order to kill malignant cells. Tumors or bystander cells such as macrophages may, however, upregulate PD-L1 and therefore obstruct T cell function by inducing inhibitory intracellular signaling. Anti-CTLA-4 blocking antibody may therefore restore T cell priming in the lymph nodes, and the PD-1 signaling blockade may enable T cell effector function at the tumor site. Additionally, other cell types such as Breg cells and DCs in the tumor microenvironment may express PD-1 and therefore be affected by PD-1 blockade. PD-1 and CTLA-4 blockade may also affect T helper cell profiles directly or by influencing the microbiota.

The direct immunological consequences of anti-PD-1 and anti-CTLA-4 treatments have mostly been investigated in T cells (Figure 2). It is thought that the blockade of CTLA-4 most likely impacts the stage of T cell activation in the draining lymph nodes when CTLA-4 expressing Tregs remove CD80/CD86 from the surface of antigen-presenting cells, thereby reducing their ability to effectively stimulate tumor-specific T cells (24). CTLA-4 blockade may also take effect at the tumor site as exhausted CTLA-4-expressing T cells and Tregs can accumulate within the tumor microenvironment (29, 53). PD-1-expressing tumor-infiltrating T cells can be disabled by PD-L1 on the surfaces of tumor cells or other infiltrating immune cells, and blocking antibodies targeting PD-1 signaling are therefore thought to mainly affect the effector stage of the immune response (13, 55–57). Since other cell types (such as dendritic cells and B cells) can also be influenced by PD-1 signaling, inhibition of the PD-1/PD-L1 pathway may also have T cell-independent effects, whose impact on immune responses during checkpoint inhibitor therapy remain to be elucidated (36, 58).

Type I immune responses, which include IFN-γ production and cytotoxic T cell functions, are important for effective anti-tumor immune responses and are associated with better responses to anti-CTLA-4 and anti-PD-1 treatments. Indeed, mouse models have shown that local IFN-γ upregulation is essential for anti-PD-1-mediated tumor regression (89). Similarly, IFN-γ and the cytotoxic granule component granzyme B were increased in regressing lesions of melanoma patients after anti-PD-1 treatment (90). Tumors in patients treated with anti-PD-1 who initially responded and then relapsed showed mutations that caused a subsequent loss in MHC class I surface expression (to avoid cytotoxic T cell recognition) or in IFN-γ response elements (6). Th9 CD4^+^ T cells have also been suggested to play a role according to a recent study that detected a significant increase in Th9 cell frequency in patients responding to anti-PD-1 treatment (91, 92).

It may be tempting to speculate that immune checkpoint inhibitors specifically boost the function of T cells belonging to the effector memory compartment, as these cells readily express cytotoxic molecules such as perforin and granzyme B. However, these cells lack the co-stimulatory receptor CD28 through which both PD-1 and CTLA-4 inhibit T cell function (93). Two recent studies have shown that it is indeed CD28-expressing cells rather than already terminally differentiated effector cells that respond to PD-1 blockade with a proliferative burst and differentiation (94, 95).

The characteristics of a tumor itself may also influence immune checkpoint inhibitor efficacy. The mutational burden of tumor cells may increase their antigenicity but may also enhance their ability to evade treatment-induced immune responses. Indeed, a recent study identified a melanoma gene signature associated with innate anti-PD-1 resistance, which included upregulation of genes associated with angiogenesis, wound healing, mesenchymal transitioning, cell adhesion, and extracellular matrix remodeling (96).

Commensal bacteria may also play a role in influencing the efficacy of immune checkpoint inhibitors. Anti-CTLA-4 treatment was found to be ineffective in mice reared under sterile conditions and to induce a shift in the gut flora of conventionally reared mice. Further experiments showed that the presence of certain bacterial strains, in particular *Bacteroides fragilis*, promoted Th1 polarization in the animals and was associated with an improved anti-tumor immune response (97). Importantly, antibiotic treatment was also associated with reduced responses to anti-PD-1/PD-L1 treatments in cancer patients, possibly by altering the normal gut flora. Good treatment response among patients was instead associated with the presence of the commensal *Akkermansia muciniphila*, which also improved anti-PD-1 treatment responses in mice by allowing increased recruitment of CCR9 + CXCR3 + CD4 + T lymphocytes into the tumor (98).

## Treatment-Related Adverse Events and Their Management

PD-1 and CTLA-4 prevent autoimmunity and limit immune activation to prevent bystander damage under physiological conditions. Inhibition of these receptors through therapeutic antibodies for the treatment of cancer is therefore associated with a wide range of side effects that resemble autoimmune reactions. Rates of severe side effects vary greatly by study and treatment (see Table 2). Clinical trials that directly compared different types of immune checkpoint inhibitors and their combination noted that more patients experienced side effects when treated with anti-CTLA-4 (27.3%) compared to anti-PD-1 (16.3%). Even more patients were affected when treated with a combination of both (55%) (67).

Almost all patients treated with immune checkpoint inhibitors experience mild side effects such as diarrhea, fatigue, pruritus, rash, nausea and decreased appetite. Severe adverse reactions include severe diarrhea, colitis, increased alanine aminotransferase levels, inflammation pneumonitis, and interstitial nephritis (67, 73, 99). There have also been reports of patients experiencing exacerbation of pre-existing autoimmune conditions such as psoriasis (91, 92, 100) or developing new ones such as type 1 diabetes mellitus (101). Particularly severe side effects may require cessation of treatment, although these patients may still respond thereafter (102). Interestingly, certain treatment-related auto-immune reactions such as rash and vitiligo have been shown to correlate with better disease prognosis (103), suggesting an overlap between auto-immune and anti-tumor immune responses.

## Biomarkers of ANTI-PD-1/CTLA-4 Treatment Efficacy

Biomarkers are needed both before and during treatment to identify the patients most or least likely to respond to immune checkpoint inhibitor treatments in order to reduce inappropriate drug exposure. Treatment response is defined as a reduction in tumor size during the course of treatment. A number of factors associated with disease prognoses in untreated patients are also linked to immune checkpoint inhibitor response rates (Table 3). For example, patients with smaller tumors or low serum lactate dehydrogenase (LDH) levels at baseline have a better prognosis and are also more likely to respond to anti-PD-1 treatment (66). A reduction in LDH levels after treatment is also associated with improved response (104). Circulating tumor DNA (ctDNA), which contains melanoma-associated mutations and can be released by dead tumor cells, can be detected in the serum of some patients. CtDNA levels correlate strongly with tumor burden and progression (105, 106). A recent study in advanced stage melanoma patients treated with anti-PD-1 (alone or in combination with anti-CTLA-4) showed high treatment response rates in individuals that were ctDNA negative prior to or after treatment (107), making serum ctDNA an attractive biomarker before and during immune checkpoint treatment.

**Table 3:** Biomarkers associated with favorable responses to immune checkpoint inhibitors.

	Pre-treatment	Post-treatment
Tumor	Tumor size and distribution (66)	Reduction in tumor size
	High mutation burden but no innate anti-PD-1 resistance (IPRES) gene signature (78, 86, 87, 96)	
	PD-L1 expression on tumor cells (only confirmed by some but not all studies) (67, 108)	

Tumor-infiltrating immune cells	Presence of CD8 + T cells inside the tumor or at the tumor margin (88)	Proliferation of intratumoral CD8 + T cells (88)
	PD-L1 expression by infiltrating cells (77)	
	Increased Th1- and CTLA-4-associated gene expression (77).	

Circulation	High relative lymphocyte counts (109)	Increased levels of ICOS + T cells (110)
	High relative eosinophil counts (109)	Low neutrophil-to-lymphocyte ratio (110)
	High serum TGF-β levels (91, 92)	High levels of Th9 cells
	Low serum LDH levels (66, 109)	A reduction in serum LDH levels (104)
	Low levels of ctDNA (107)	A reduction in ctDNA (107)

Host genome	Presence of HLA-A*26 allele (111)	

For anti-PD-1 treatments, expression of PD-L1 within the tumor microenvironment has been an obvious biomarker candidate. Although PD-L1 expression on tumor cells was correlated with treatment efficacy in melanoma patients (67, 108), it was not in patients with squamous cell carcinoma, non-small cell lung cancer and Merkel cell carcinoma (70, 72, 74). Interestingly, one study assessing the role of PD-L1 in both cancer cells and tumor-infiltrating immune cells found that only in the latter context was anti-PD-L1 treatment efficiency correlated with PD-L1 expression (77).

The presence of neoantigens on mutated tumor cells boosts anti-tumor immunogenicity and improves treatment efficacy. High genetic disparity between tumor cells and host cells is therefore an indicator of checkpoint inhibitor treatment efficacy. This was particularly noted in anti-CTLA-4-treated melanoma patients whose tumors displayed neo-antigens (87) and similarly in anti-PD-1-treated patients with colorectal cancers or non-small cell lung cancers that were mismatch-repair deficient or had high mutation rates, respectively (78, 86). Although overall mutational burden is associated with improved response to anti-PD-1 treatment, reduced responses were detected in melanoma patients whose tumors displayed the IPRES gene signature (96). Antigen presentation by the host may also play a role during anti-PD-1 treatment, as patients with the HLA-A*26 were more than twice as likely to respond than patients negative for the allele (111).

Other pre-treatment immunological factors associated with improved treatment responses include high eosinophil and lymphocyte blood counts, an abundance of CD8^+^ T cells infiltrating the tumor or present at the tumor margin, and increased serum TGF-β levels in melanoma patients treated with anti-PD-1 (88, 91, 92, 109). Increased Th1 and CTLA-4 (but not FoxP3) gene expression levels were also noted in responder patients with various solid tumors (including melanoma) treated with anti-PD-L1 (77).

A number of post-treatment immunological observations have also been associated with improved immune-checkpoint inhibitor responses. For example, patients more likely to respond to anti-CTLA-4 treatment had increased numbers of inducible co-stimulatory molecule (ICOS) expressing T cells and lower neutrophil-to-lymphocyte ratios (110). An increase in CD8^+^ T cell proliferation within the tumor lesion and an increased frequency of Th9 cells in the patients’ circulation were also associated with treatment response (88, 91, 92).

Taken together, many of these studies indicate that immune checkpoint inhibitors are most effective in patients who already display anti-tumor immune processes prior to therapy. However, not all biomarkers listed here may be equally effective, and patients may still respond to treatment despite contrary biomarker-based predictions. Further, accessing tumor tissue may be difficult in many patients, especially after treatment, and less invasive blood-based “liquid biopsies” may therefore be more appropriate. Importantly, it has been shown that investigating several biomarkers in combination can improve treatment predictions (109). Although the recently discovered ctDNA seems to be a particularly promising biomarker candidate, more studies are needed to identify more effective biomarkers or biomarker combinations, in order to devise the most appropriate treatment strategy for each patient.

## Limitations of Immune Checkpoint Inhibitors

Although immune checkpoint inhibitor treatment may be effective initially, many patients will eventually relapse and develop tumor progression. A number of studies have therefore sought to understand the mechanisms by which anti-PD-1 and anti-CTLA-4 treatments lose their efficacy.

The selection pressure caused by checkpoint inhibitor treatment may give rise to tumor cells that can evade immunomediated recognition and deletion through new pathways. Tumor cells from patients refractory to anti-PD-1 treatment, for example, were recently shown to have acquired mutations making them less susceptible to T cell-mediated killing *via* loss of IFN-γ response elements or MHC class I (6).

Anti-PD-1 or anti-CTLA-4 treatment may also cause upregulation of other inhibitory receptors. For example, patients with melanoma or prostate cancer exhibited upregulation of the inhibitory receptor V-domain Ig suppressor of T cell activation (VISTA) on various tumor-infiltrating immune cells after anti-CTLA-4 treatment (112). Another study noted the upregulation of the inhibitory receptor TIM-3 (but not VISTA) on the surface of T cells in anti-PD-1-treated mice with lung cancer as well as TIM-3 upregulation on T cells in adenocarcinoma patients refractory to PD-1 treatment (113).

Most recently, a study revealed another unexpected resistance mechanism to anti-PD-1 therapy in mice whereby tumor-associated macrophages removed the therapeutic antibody from the surface of the T cells *in vivo*, thus making them once again susceptible to inhibitory signaling through the receptor. This phenomenon could be partially overcome by administration of Fc-receptor blocking agents prior to treatment (114). A better understanding of the mechanisms limiting the effectiveness of immune checkpoint inhibitors will therefore allow improvement of future treatments.

## Future Avenues: Expanding the Immune Checkpoint Inhibitor Treatment Repertoire

PD-1 and CTLA-4 blocking agents are not effective in all patients, and even those patients who do respond initially can relapse, highlighting the need for improved or alternative treatments. Alternative inhibitory receptors have been identified that may also be targeted for anti-tumor immune therapy. These include the TIM-3, LAG-3, TIGIT, and B- And T-Lymphocyte-Associated Protein (BTLA) receptors associated with T cell exhaustion as well as VISTA, a receptor found on tumor-infiltrating myeloid cells, whose inhibition promoted anti-tumor immune responses in murine models, and CD96, which has been shown to inhibit NK cell activity in murine cancer models (115–117).

Combinations of immune checkpoint inhibitors with each other or with other treatments are also being explored. Indeed, the combination of anti-CTLA-4 with anti-PD-1 treatments showed superior efficacy compared to individual administration, but was also associated with an increase in side effects. The tryptophan-metabolizing enzyme IDO inhibits T cell function, and combining IDO-blocking agents together with immune checkpoint inhibitors has shown promising results in mice and is also currently undergoing clinical trials in humans (105, 118). Macrophages may also interfere with anti-tumor immunity or even directly restrict therapeutic antibodies (114). Their depletion through a Colony stimulating factor-1 receptor (CSF-1R) inhibitor is therefore being explored in clinical trials together with anti-PD-1, after having shown efficacy in a glioblastoma mouse model (119). Anti-tumor T cell function induced by PD-1 blockade in mice could also be improved by a targeted increase in mitochondrial function (120).

Because immune checkpoint inhibitors work by removing brakes on the immune system rather than directly boosting immune function, patients may also benefit from combination therapies that include immunostimulatory substances. Mouse melanoma models, for example, have shown that the combination of anti-CTLA-4 with cytokines such as granulocyte-macrophage colony-stimulating factor (GM-CSF) or with agonistic antibodies targeting costimulatory receptors such as CD40, increased tumor rejection in a synergistic manner (121, 122). The genetically modified herpes simplex virus talimogene laherparepvec is designed to replicate in tumor cells and to release GM-CSF, thus attracting immune cells into the tumor environment. The virus has been tested in recent clinical trials in combination with either CTLA-4 or PD-1 in advanced-stage melanoma patients, resulting in increased treatment response rates compared to the immune checkpoint inhibitors alone (123, 124).

Even modulation of the gut microbiome may improve immune checkpoint inhibitor-based therapies. Administration of intestinal *Bifidobacteria* alone was associated with reduced tumor growth in a murine B16 melanoma model by promoting dendritic-cell mediated CD8^+^ T cell responses. Importantly, the administration of these bacteria also added to the therapeutic effect of anti-PD-1 treatment in these mice (125). In a similar study, administration of *B. fragilis* to sterile mice treated with anti-CTLA-4 resulted in reduced tumor growth, most likely by inducing a favorable shift toward Th1 responses (97). Studies in humans were further able to link the presence of fecal *A. muciniphila, Ruminococcaceae*, and *Faecalibacterium* to a favorable outcome to anti-PD-1 treatment (98, 126) Together, these findings suggest that human patients too may benefit from appropriate management of their intestinal flora while undergoing immune checkpoint inhibitor treatment.

A wide range of promising new avenues are therefore currently being explored, although their clinical efficacy remains to be confirmed by ongoing and future clinical trials.

## Concluding Remarks

Although PD-1 and CTLA-4 targeting therapies have been able to increase average life expectancy for cancer patients, mortality remains high among advanced-stage patients, highlighting the need for further innovation in the field. Both anti-PD-1 and anti-CTLA-4 therapies appear to be more effective in patients with pre-existing anti-tumor immunity, suggesting that, in patients without such immunity, these drugs are unable to mediate anti-tumor immune responses *de novo*. However, as our understanding of the mechanisms of these drugs improves, avenues are being opened to improve their use not only by specifically targeting those patients who are most likely to respond through appropriate biomarker screening procedures, but also by pairing currently used immune checkpoint inhibitors with other complimentary drugs to help those patients unable to respond to the current regimens.

## Author Contributions

This review was drafted by JAS and AO, and critically revised by KK.

## Conflict of Interest Statement

AO has been awarded research grants by ONO PHARMACEUTICAL CO., LTD. and Bristol-Myers Squibb.

## Abbreviations

ctDNA, circular tumor DNA; CTLA-4, T-lymphocyte-associated protein 4; DCs, dendritic cells; IPRES, innate anti-PD-1 resistance; LDH, lactate dehydrogenase; NK, natural killer cells; PD-1, programmed cell death protein 1; IDO, indoleamine 2,3-dioxygenase; IL-12, interleukin 12; TGF-β, tumor growth factor-β; Tregs, regulatory T cells; MDSCs, myeloid-derived suppressor cells; VISTA, V-domain Ig suppressor of T cell activation; ITIM, immunoreceptor tyrosine-based inhibition motif; IFN-γ, interferon; TIM-3, T-cell immunoglobulin and mucin-domain containing-3; LAG-3, lymphocyte-activation protein 3; TIGIT, T-cell immunoreceptor with Ig And ITIM domains; TNF-α, tumor necrosis factor-α; ICOS, inducible co-stimulatory molecule; IFN-γ, interferon-γ; BTLA, B- And T-lymphocyte-associated protein; CSF-1R, colony stimulating factor-1 receptor; GM-CSF, granulocyte-macrophage colony-stimulating factor; Breg, regulatory B cell.
